# Evaluating the effectiveness of drones for quantifying invasive upside-down jellyfish (*Cassiopea* sp.) in Lake Macquarie, Australia

**DOI:** 10.1371/journal.pone.0262721

**Published:** 2022-01-19

**Authors:** Claire E. Rowe, Will F. Figueira, Brendan P. Kelaher, Anna Giles, Lea T. Mamo, Shane T. Ahyong, Stephen J. Keable

**Affiliations:** 1 School of Life and Environmental Sciences, University of Sydney, Sydney, New South Wales, Australia; 2 Marine Invertebrates, Australian Museum Research Institute, Sydney, New South Wales, Australia; 3 National Marine Science Centre, Southern Cross University, Lismore, New South Wales, Australia; 4 School of Biological, Earth and Environmental Sciences, University of New South Wales, Kensington, New South Wales, Australia; CSIR-National Institute of Oceanography, INDIA

## Abstract

Upside-down jellyfish (*Cassiopea* sp.) are mostly sedentary, benthic jellyfish that have invaded estuarine ecosystems around the world. Monitoring the spread of this invasive jellyfish must contend with high spatial and temporal variability in abundance of individuals, especially around their invasion front. Here, we evaluated the utility of drones to survey invasive *Cassiopea* in a coastal lake on the east coast of Australia. To assess the efficacy of a drone-based methodology, we compared the densities and counts of *Cassiopea* from drone observations to conventional boat-based observations and evaluated cost and time efficiency of these methods. We showed that there was no significant difference in *Cassiopea* density measured by drones compared to boat-based methods along the same transects. However, abundance estimates of *Cassiopea* derived from scaling-up transect densities were over-inflated by 319% for drones and 178% for boats, compared to drone-based counts of the whole site. Although conventional boat-based survey techniques were cost-efficient in the short-term, we recommend doing whole-of-site counts using drones. This is because it provides a time-saving and precise technique for long-term monitoring of the spatio-temporally dynamic invasion front of *Cassiopea* in coastal lakes and other sheltered marine habitats with relatively clear water.

## Introduction

Jellyfish populations are highly variable and many species can grow and reproduce rapidly in response to favourable environmental conditions [1]. Enhanced dispersal from vessel ballast water, biofouling and aquaculture releases are each increasing the potential for jellyfish to thrive and expand their range [2–5]. Furthermore, anthropogenic stressors, such as overfishing and habitat degradation, create openings for the opportunistic colonisers [2,6]. Many species of jellyfish are considered as ‘cryptogenic’ because little is known about their native range, and accurate estimates of their population size, biomass, distribution, and movements are challenging to obtain [7–10]. There is, therefore, limited data on historical abundances of jellyfish, and this has been exacerbated by past biases about their relative importance to marine ecosystems [11,12]. However, jellyfish are both ecologically and commercially significant (e.g. carbon sequestration from their falls or fisheries), so it is important to understand their distribution and population dynamics [13–15].

Upside-down jellyfish (*Cassiopea* spp.) are found globally in tropical and sub-tropical regions, in shallow, protected habitats such as coral reefs, mangrove forests and seagrass beds [16–18]. These jellyfish exhibit sedentary behaviour with their bell downwards on the sediment and oral arms extending upwards into the water column [16,19]. *Cassiopea* can alter the structure and function of ecosystems through direct impacts, including predation on zooplankton such as fish larvae, and indirect impacts, including displacement of local fish by the release of stinging cells into the water column [13,20]. *Cassiopea* stings can also be harmful to humans [13,21]. *Cassiopea* are successful invaders because of their high tolerance to environmental variation and their ability to rapidly reproduce sexually and asexually in large numbers, as well as lie dormant as polyps [19,22–24]. There are a number of recent first records of *Cassiopea* as they expand their distributional range in the Mediterranean, as well as Hawaiian and Australian waters [16,19,25–27].

To date, there are only quantitative reports of the abundance of the medusa (i.e. adult) stage of *Cassiopea* at a global scale, and these have mostly used quadrat sampling at a single point in time [28–31]. Owing to boom-and-bust dynamics and patchiness of *Cassiopea* populations, abundance estimates from quadrat sampling often lack precision, which makes it challenging to track invasion dynamics. There is, therefore, a need for more cost-effective monitoring techniques that provide precise estimates of *Cassiopea* abundance over management-relevant spatial and temporal scales.

Remotely piloted aircraft (hereafter called drones) are becoming increasingly popular for environmental [32] and wildlife monitoring [33–35]. The advantages of using drones over direct field observations are that they can reduce survey time and cost, and increase the accuracy of surveying a large area [36]. Drones have been effectively used to monitor pelagic jellyfish, such as *Catostylus mosaicus* [36], *Aurelia* sp. [10] and *Chironex fleckeri* [37], and to detect the presence of *Cassiopea* in aquaculture systems [38]. However, the use of drones for long-term monitoring of sedentary *Cassiopea* in their natural environment has not yet been explored. Here, we evaluate the utility and efficiency of drones for estimating the density of *Cassiopea* medusae by comparing results of drone-based monitoring to a conventional direct observation from a kayak.

## Methods

### Data collection

#### Field sampling

This study was undertaken at Lake Macquarie (33°04’00”S, 151°32’42”E), a coastal estuary on the east coast of Australia. The water temperature in the area ranges from 33°C in summer, down to 12°C in winter [39]. In Lake Macquarie, *Cassiopea* were first observed in early June 2017 in depths of less than 2 m [25], and subsequently verified shortly after, with multiple specimens collected under Department of Primary Industries Scientific Collection Permit Number: F86/2163-8.0 and deposited in the collections of the Australian Museum (registrations G.18362 to G.18365). Genetic and morphological comparisons indicate it is the same species that has previously been recorded 150 km to the north in Wallis Lake (32°15’57”S, 152°29’08”E) and so the occurrence of *Cassiopea* in Lake Macquarie appears to be a further southward range-expansion [16].

**Fig 1:**
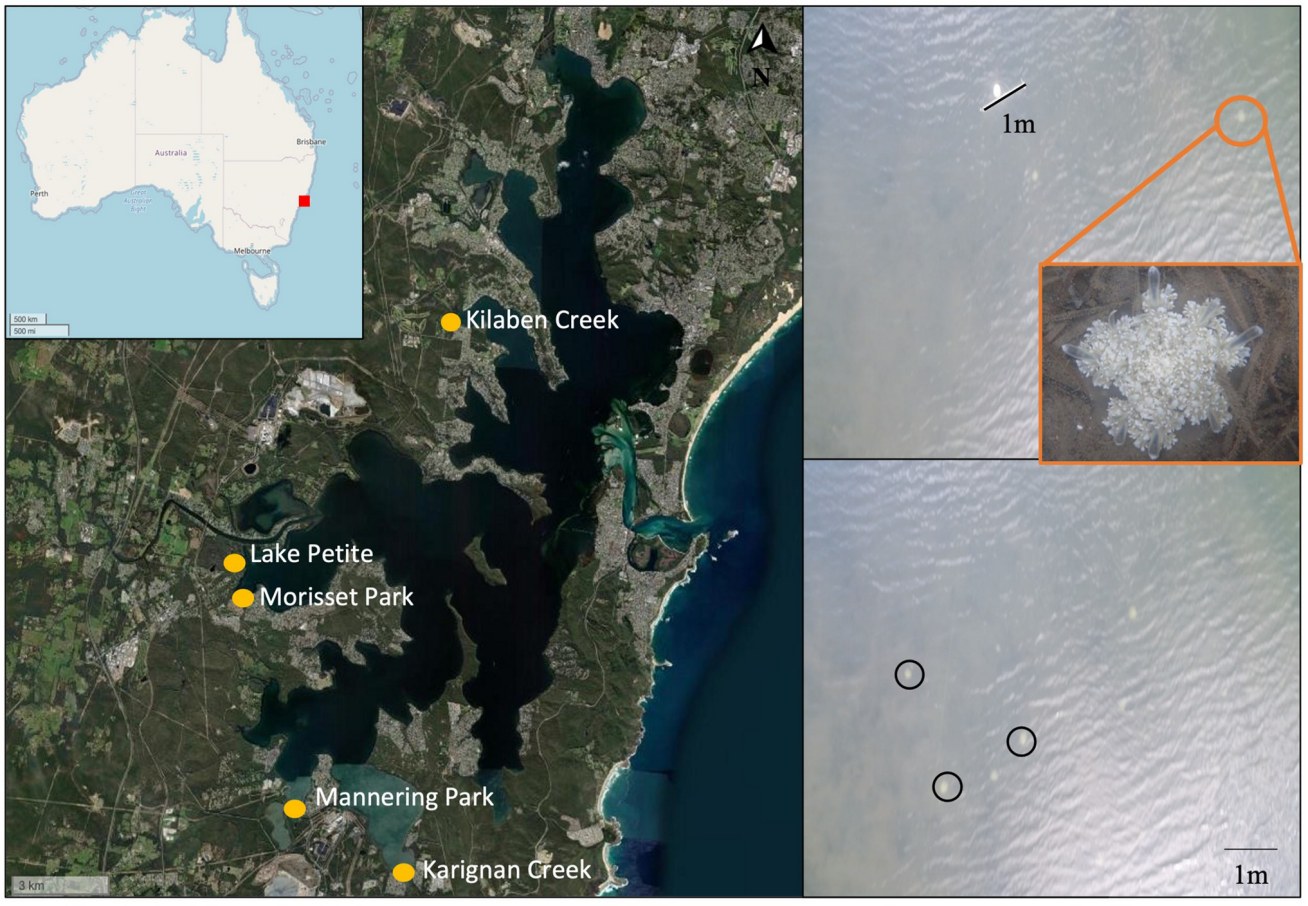
Study locations around Lake Macquarie, Australia. A) shows buoy and 1m PVC pipe at the end of the transect, which was used for scale. B) shows a cluster of *Cassiopea* with black circles around 3 individuals. Aerial images used for A) and B) were taken from a drone at an altitude of 6.7m. Maps were generated using the leaflet package in R (base map data from OpenStreetMap and OpenStreeMap Foundation under the CC BY-SA 2.0 License).

Field sampling was undertaken on 27 and 29 May 2019 at five sites (Fig 1) within Lake Macquarie. Each site included a large embayment of the lake, averaging (± SE) 22,459 ± 7,541 m^2^ in area. Within each site, three 50 m transects were randomly marked along the 60 cm depth contour, running parallel to the shore. The start and end of transects were marked with buoys and 1 m PVC pipe for scale. The transects did not go deeper than 60 cm because *Cassiopea* mostly occur in shallow water environments and can reliably be detected at this depth [17,29,31,40]. A secchi disc measurement in the middle of each site was used to assess relative water clarity and visibility.

#### Direct observations

A kayak was utilised to access shallow, muddy areas that cannot be easily traversed on foot. For direct field observations from the kayak, the observer counted the number of *Cassiopea* along the 1 m wide transects. It was important to determine variation in *Cassiopea* densities as a function of depth and distance from shore, because the depth at which either technique would work was poorly understood, and because drones cannot effectively sample deep water or close to shore due to mangrove cover (see Methods for detailed analysis of sites). For this reason, the number of *Cassiopea* along depth stratified transects at 15, 30 and 60 cm depth were observed using the same direct observation method from the kayak (see above). This was completed as part of a broader study investigating the population dynamics of *Cassiopea* within Lake Macquarie between 6 and 9 May 2019 at the same sites around Lake Macquarie with the exception of Mannering Park (due to difficulty accessing the site from kayak, Rowe, unpublished data, Fig 1).

#### Drone observations

Drone surveys were carried out with a DJI Phantom 4 Pro drone with 2.54 cm CMOS 20 MP sensor with ND8 polarising filter and positioned in nadir. The drone surveys were undertaken when wave action and surface reflection were sufficiently low to enable *Cassiopea* to be observed throughout the 60 cm water column [36,41]. Drones were flown at an altitude of 6.7 m above the water’s surface, which provided a 10 m wide field of view. Drones were flown along the same transects used by the kayak by pinpointing the buoys at either end of each transect and automating a path between them. Additionally, the program Litchi was used to design and fly in a computer-controlled grid, recording evenly spaced video transects at a 6.7 m altitude, designed to capture the entirety of each site. This method was used to count the number of *Cassiopea* detected across the full site and will be referred to as a ‘whole-of-site count’. The carefully spaced grid ensured that the entire site was sampled and no *Cassiopea* double counted.

### Data analysis

#### Analysis of transects

Drone footage from each transect was analysed for presence of *Cassiopea* in Kinovea (v 0.8.15) (Fig 1). The images were scaled using the 1 m PVC pipe, and the number of *Cassiopea* along the 50 x 1 m transect was recorded (Fig 1). The counts from direct observation and drone footage were used to calculate the density of *Cassiopea* per meter square for each transect. To test the hypothesis that density estimates between direct and drone observations differed significantly, a two-way repeated measure ANOVA was used, including the factors “method” (fixed; two levels, drone and direct) and “site” (random; 5 levels). All statistical analyses were completed in R (V.3.6.3) [42]. The model assumptions were tested using Levene’s test for homoscedasticity and Shapiro-Wilk’s test for normality. If either assumption was violated, a natural log transformation was used, after which assumptions were satisfied. The relative precision (P) of counts from drone footage compared to direct visual observations from a kayak was estimated using Standard Error (SE)/mean [34,43] of transects for each method at each site.

Any potential effect of turbidity on the relationship between *Cassiopea* abundance estimates for each of the two surveying techniques was assessed using a multiple linear regression, with drone footage density estimates as the response variable and direct observation density estimates and the secchi depth reading of each site as explanatory factors. Multiple regression assumptions were tested including linearity, independence, normality, homoscedasticity, and multicollinearity.

#### Analysis of sites

The objective of the site-level analysis was to compare scaled-up kayak and drone transect estimates with a whole-of-site drone count. However, important variables considered a-priori were: the drone could not effectively survey areas close to shore due to the overhang of mangroves interfering with the drone’s visual area, and the depth to which either technique would reliably detect *Cassiopea* was relatively unknown. To overcome these constraints, we first conducted an analysis to determine if there was any evidence that *Cassiopea* densities were influenced by depth or distance from shore in order to revise the survey area if required. We then restricted the area of interest for the scaled-up estimates to the depth where both techniques would be able to identify *Cassiopea* reliably. A conservative maximum depth of 60 cm was selected based on the congruence between drone and direct observation counts at this depth in the transect-level analysis (see Results), and two years’ survey experience in these waters under conditions of variable water quality (C. Rowe, pers. observations).

In order to determine the variation of *Cassiopea* at different depths, a Kruskal-Wallis H analysis was used to test whether the density of *Cassiopea* varied between depth stratified transects, followed by a pairwise comparison using Wilcoxon signed-rank test. Additionally, a Kruskal-Wallis H analysis was used to ensure there was no difference in the density of *Cassiopea* between sites. A distance from shore analysis was conducted by measuring the distance from shore of each depth stratified transect using GPS waypoints in ArcMap (V.10.7). A general linear model was used to determine if there was a relationship between the density of *Cassiopea* along these transects and their distance from shore and if this was influenced by site. This confirmed there was a significant difference in the density of *Cassiopea* among the depth of the stratified transects with a greater number of *Cassiopea* occurring at 15 cm depth compared to 30 or 60 cm, but there was no relationship between density and distance from shore (see Site-level results).

As there was no significant relationship with distance from shore, and due to the shallow nature of *Cassiopea* habitat, the area sampled by the whole-of-site drone count was compared to *Cassiopea* densities recorded from the drone and kayak transects multiplied by the area of each site calculated to be equal or less than 60cm. This was calculated by using bathymetry data sourced from Australia’s Integrated Marine Observing System (IMOS) in ArcMap (V.10.7). Transect estimates confined to the area of each site that was equal to or less than 60cm were compared to the number of *Cassiopea* counted in the whole-of-site drone count using a one-way repeated measure ANOVA, where “method” was the repeated measures factor, having estimates from each method at each of the five sites. To compare the three methods of quantifying the density of *Cassiopea* for each site, 95% confidence intervals were used, comparing whole-of-site drone count with the scaled-up transect-based estimates using kayak and drone data. The lower- and upper-bound 95% confidence intervals were calculated for the mean of each technique, transect and site, and then scaled according to the area that was ≤ 60 cm depth for that site. Homoscedasticity and normality for the site-level analysis were tested using similar method to those described above.

To determine the time-efficiency of drones for a whole-of-site count of *Cassiopea* compared to visual observations along transects using a kayak, the time taken to complete surveys by each method was recorded.

## Results

### Transect-level results

**Fig 2:**
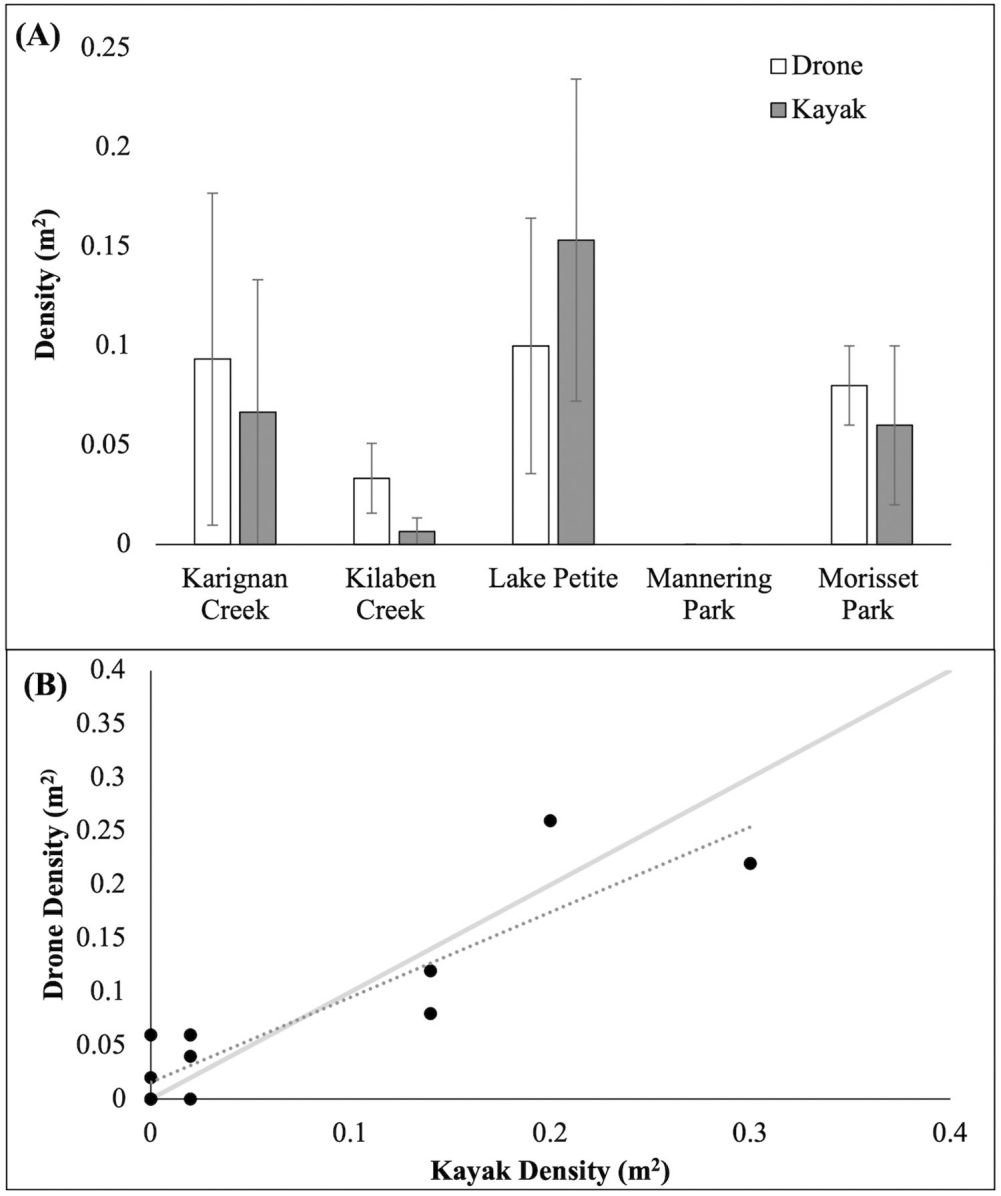
A) The mean density of *Cassiopea* (m^2^) that were detected by a drone compared to visual observations made in a kayak. Error bars are ± SE. B) The density of *Cassiopea* detected by a drone (m^2^) compared to the density detected by visual observation in a kayak (m^2^) at a transect level across 5 sites. The grey dashed line indicates the line of best fit. The grey solid line indicated the 1:1 ratio of the density detected by drones compared to kayak.

There was no significant difference in *Cassiopea* density estimates derived from the two survey techniques (F_1,8_ = 0.429, *p* = 0.580, Fig 2A), nor was there any effect of site (F_4,8_ = 1.207, *p* = 0.370) or an interaction with method (F_4,8_ = 4, *p* = 0.138). The mean (± SE) density per m^2^ of *Cassiopea* that were detected along transects by drones (0.0613 ± 0.021) was 6.5% greater than visual observations made from a kayak (0.0573 ± 0.024). Across all sites, except for Lake Petite, drones had a higher relative precision (lower P indicating higher precision, Table 1) compared to visual observations made from a kayak. The relative precision of drones ranged between 0.25 and 0.89 across all sites, while the use of a kayak ranged between 0.53 and 1.0. At Mannering Park, no *Cassiopea* were observed by either drone or kayak during the study period although they have been found in this locality previously (C. Rowe, pers. observations).

**Table 1:** Precision (P = Standard Error (SE)/mean) of the density of *Cassiopea* detected by drones compared to visual observations from a kayak. A lower P value indicates a higher precision in detection of *Cassiopea*. ‘No obs.’indicates that no *Cassiopea* were observed using that method at that site.

Site	Drone P	Kayak P
Karignan Creek	0.89	1
Kilaben Creek	0.53	1
Lake Petite	0.64	0.53
Mannering Park	No obs.	No obs.
Morisset Park	0.25	0.67

The multiple regression model indicated that *Cassiopea* density estimated by kayak and drone observations were significantly associated (F_2,14_ = 32.310, *p* < 0.001, R^2^ = 0.843, slope = 0.107; kayak-t_(14)_ = 7.770, *p* < 0.001, Fig 2B), but there was no effect of water clarity (t_4_ = -1.285, *p = 0*.*223*).

### Site-level results

**Fig 3:**
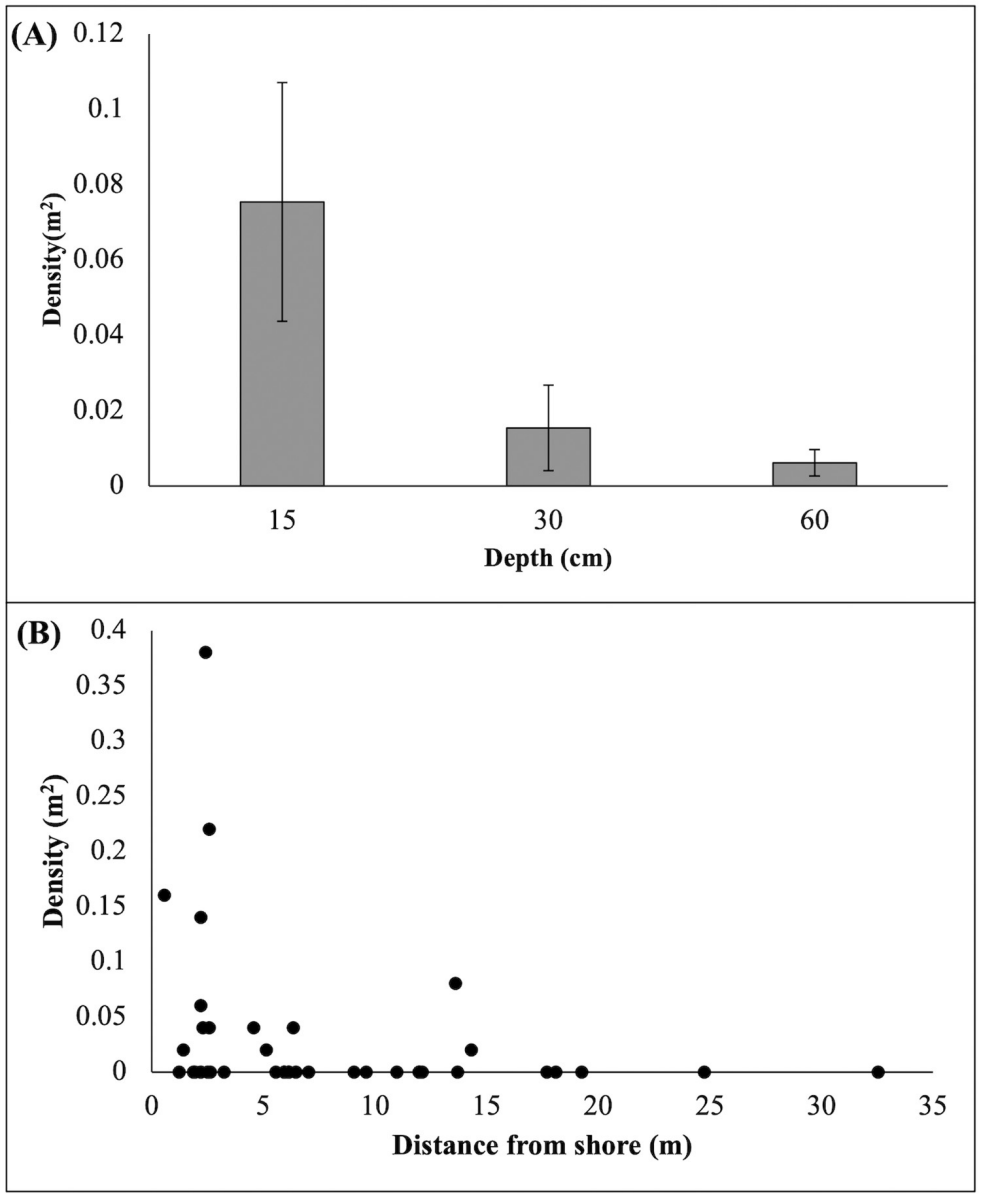
A) The mean density of *Cassiopea* (m^2^) occurring at 15, 30 and 60 cm depth in Lake Macquarie. Error bars are ± SE. B) Density of *Cassiopea* (m^2^) as a function of distance from shore across 4 sites in Lake Macquarie, NSW.

There was a significant difference in the density of *Cassiopea* occurring along the different depth stratified transects (X^2^(2) = 7.892, *p < 0*.*05*, Fig 3A) with a significantly greater mean density (± SE) per m^2^ of *Cassiopea* found along the 15 cm transect (0.075 ± 0.032) compared to 30 cm (0.015 ± 0.011, *p < 0*.*05*) or 60 cm (0.006 ± 0.003, *p < 0*.*05*) depth, which did not differ significantly (*p = 0*.*823*). Additionally, there was no significant difference in the density of *Cassiopea* occurring between sites (X^2^(3) = 0.805, *p = 0*.*848*). While most *Cassiopea* individuals appeared to be less than 15 m from shore, the general linear model was not significant (F_2,36_ = 1.774, *p = 0*.*184*, R^2^ = 0.09, Fig 3B), indicating no effect of distance from shore on *Cassiopea* densities.

**Fig 4:**
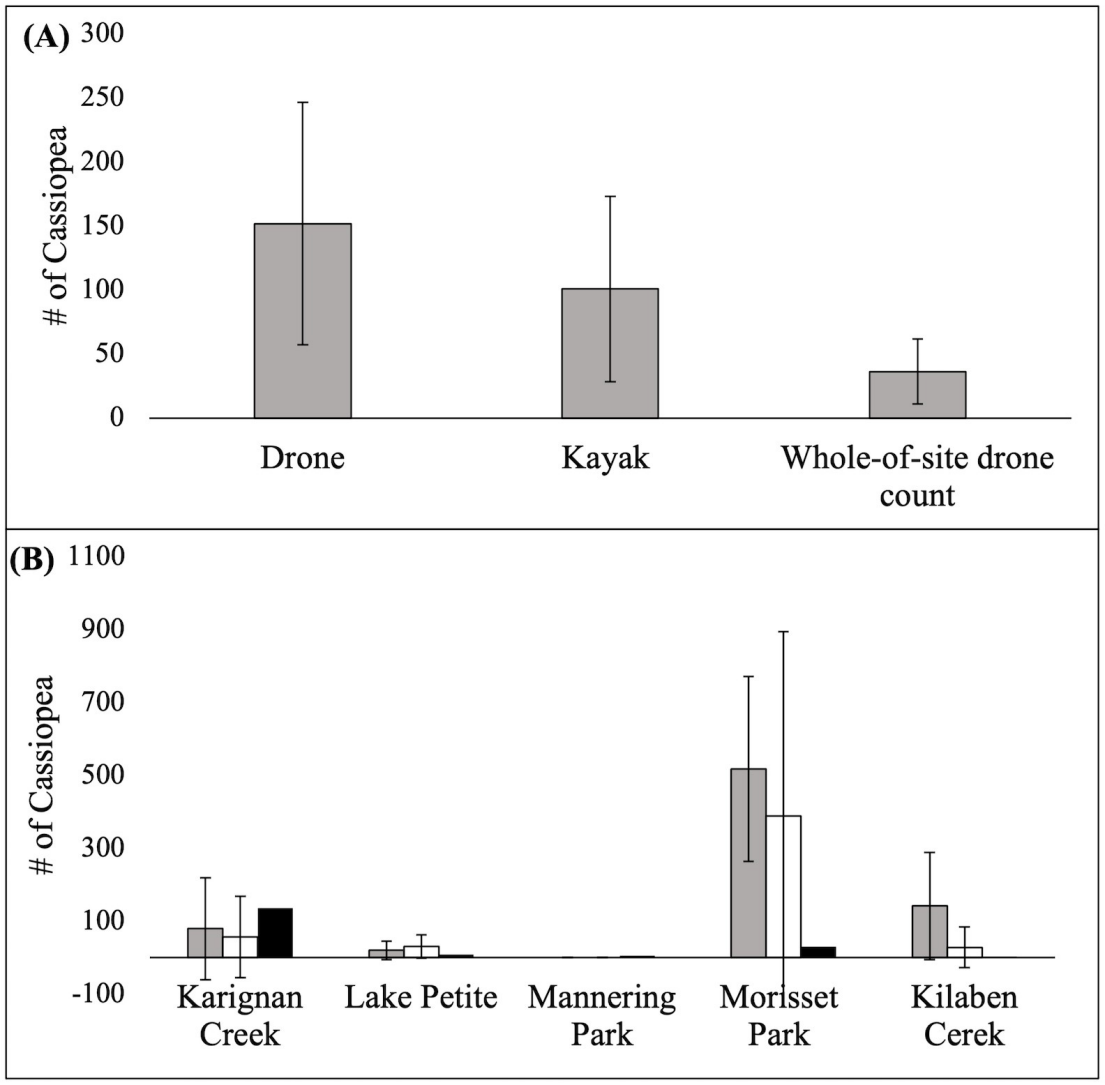
A) The mean number of *Cassiopea* estimated by the drone and kayak transects to be in ≤ 60cm compared to the mean number of *Cassiopea* the whole-of-site drone count actually detected across 5 sites. Error bars are ± SE. B) The mean number of *Cassiopea* predicted by drone (Grey) and kayak (White) transects to be in ≤ 60 cm of the whole-of-site drone count at each site compared to the actual number of *Cassiopea* detected by the whole-of-site drone count (Black). Error bars are ± 95% CI.

The mean (± SE) site-level abundance of *Cassiopea* estimated from drone (151.85 ± 94.75) and kayak transects (100.82 ± 72.41) were substantially larger than those derived from the whole-of-site drone counts across these same sites (36.2 ± 25.43), although these differences were not significant (F_2,4_ = 2.911, *p* = 0.131, Fig 4A). Lower abundance counts of *Cassiopea* using whole-of-site drone count compared to drone and kayak transect-derived estimates was found at each site, with the exception of Karignan Creek, which showed the opposite pattern (Fig 4B). However, in all cases, the whole-of-site drone count falls well within the 95% confidence intervals from the transect-based estimates.

The overall process of setting-up, sampling, packing-up and analysing the density of *Cassiopea* using visual observations made from a kayak took around 90 minutes, which is almost twice as long as completing a whole-of-site drone count, which would take around 55 minutes (Table 2). Owing to the patchy nature of *Cassiopea*, for a site averaging around 22,459 m^2^, a total of 9 transects would be required to appropriately cover the site and assess *Cassiopea* abundance using a kayak. This would take around 180 minutes, which is over three times longer than using a drone.

**Table 2:** The number of minutes estimated a drone takes to sample and analyse the number of *Cassiopea* at each site compared to the amount of time it took to take visual observations from a kayak in this study (3 transects), and the amount of time it would take to sample a full site from a kayak (we estimate this would require around 9 transects).

	Drone	Kayak (3 transects)	Kayak (9 transects)
Pre-sample set up	~10	~20	~20
Sample full site	~15	~45	~135
Post-sample pack up	~5	~15	~15
Analysis	~25	~10	~10
**Total**	**~55**	**~90**	**~180**

## Discussion

The invasive nature of *Cassiopea* and relatively inaccessible habitats in which it often occurs generates the need for a reliable, cost-effective method of recording their abundance and distribution over large spatial scales. We demonstrate that both drones and kayak-based observations are effective monitoring methods, with drones mostly averaging a higher density of *Cassiopea* detected at each site. Similar results were achieved in a study examining the use of drones for detecting pelagic jellyfish (*Catostylus mosaicus*), finding that drone footage detected more individuals than visual observations [36]. In our study, the drone altitude of 6.7 m above the surface could have allowed for more *Cassiopea* to be detected than direct observations from the surface using a kayak. However, the extra height biases the drone towards larger individuals and could miss small jellyfish (< 3 cm diameter), which occur at the start of a jellyfish bloom and which are more likely to be detected by closer observations such as those made from the kayak [44]. This is a possible explanation for the greater density of *Cassiopea* at Lake Petite detected using direct observations from a kayak, rather than drone, and is why direct observations from a kayak should be considered a proxy for the true value of the number of *Cassiopea* occurring during the start of a bloom. Monthly or even weekly assessments of *Cassiopea* would be straightforward using a drone and would allow blooms to be detected as they occur. This is important because of the high rate at which jellyfish populations expand and collapse, allowing successful precautionary methods to be developed to counteract any impacts [1,36,45].

For transects, drones had a higher precision than visual observations from a kayak across all sites. However, *Cassiopea* have patchy distributions within each site, made evident by the large 95% confidence intervals for drone and kayak transects quantifying the density of *Cassiopea* (Fig 4B). This patchiness could explain why at Karignan Creek more *Cassiopea* were detected in the whole-of-site drone count, rather than along the transects, which may have been placed on the outskirts of a bloom. The whole-of-site drone count of *Cassiopea* at each site was well within the 95% confidence intervals due to the huge variance of the transect-based estimates. For this reason, completing whole-of-site drone sampling may provide more precise estimates of the number of *Cassiopea* occurring than estimates derived from transect densities. Additionally, a drone is also more feasible for covering larger areas in a shorter time [36]. Further studies should test the maximum depth at which *Cassiopea* occur and are able to be detected by drones across a range of turbidity-levels to ensure whole-of-site drone counts are not missing any individuals deeper than 60 cm.

Drones and direct observations come with the risk of detection errors, such as availability and perception errors [46]. An availability error would be *Cassiopea* occurring in the survey area but not being visible to the drone. A perception error is when the jellyfish is visible to the drone, but is missed or misclassified as another taxon or object, or incorrectly classified as the target taxon [47–50]. Both availability errors and perception errors are influenced by environmental variables such as time of day, turbidity, sun glint, wind and cloud cover [33,46,50]. These factors also relate to the sightability of a species, which is associated with its size, morphological attributes and behaviour [48,51,52]. For our study, we do not believe turbidity caused an availability error because of the shallow water depths and because the drone detected a greater density along the transects than the kayak observations. During the survey period the average secchi disc reading across sites was 117 cm ± 0.189 cm, almost twice the maximum depth considered for observation. However, false-positive (i.e. perception) errors could have occurred by counting taxonomic groups with similar colours or morphological characteristics, such as mollusc shells, or inanimate objects such as plastic bags, golf balls and other items that were found scattered around some of our sites, potentially leading to an overestimation of the number of *Cassiopea*. Direct observation from close range is more likely to lower these perception errors as diagnostic taxon characters can be more readily distinguished, and individuals physically examined if required.

Despite these limitations, our results indicate that whole-of-site drone counts provide an acceptable benchmark to detect and record the abundance of benthic *Cassiopea* medusae in shallow environments. Future studies should georeference transects due to the featureless nature of water, allowing the size of the jellyfish bloom to be determined and its movement tracked [10]. Additionally, if associated bathymetry data is accessible, this would allow the depth of *Cassiopea* to be calculated during analysis, allowing any depth-abundance patterns to be detected and the maximum depth drones can detect *Cassiopea* to be determined. If no bathymetry data is available, an integrated approach of the two methods is recommended, with drones determining the spatial extent of jellyfish blooms, and kayaks assessing any depth-abundance patterns. The shallow water occurrence and easy detectability of *Cassiopea* provides the opportunity for automated object-based image analyses and deep learning neural networks, which will greatly improve the cost-effectiveness of drone surveys [36,50,53,54]. This has been used for pelagic jellyfish (*Catostylus*) allowing for rapid calculations of their numbers, densities, size frequencies and biomass [36]. Additionally, drones have been used to determine the number and density of *Cassiopea* in aquaculture farms [38]. However, the location of *Cassiopea* was already known and a hand-held camera was used to assist density and number calculations, which may not be feasible in natural environments or at larger scales.

Most drones are more subject to favourable weather conditions than direct observation, as they are unable to obtain useable images in winds greater than 30 km h^-1^ and during periods of heavy rainfall. Additionally, the drone often must remain in visual line of site for many civilian applications [50] and our drones were not able to fly close to obstacles, such as mangroves or other trees, common along the shoreline. This restricted the current study because there were a number of sites where *Cassiopea* were present and amenable to direct observations but not drone survey.

Our results showed that *Cassiopea* are most abundant at 15 cm depth compared to 30 cm or 60 cm. This creates potential for the whole-of-site drone count to underestimate the size of the bloom by missing jellyfish at 15 cm depth, which may be around obstacles close to shore. However, we do not believe this was the case because, while *Cassiopea* were typically found within 15 m of the shoreline, there was no linear relationship between their density and distance from shore. However, if there is a problem of obstacles close to shore restricting drone flight paths, as long as the water is still visible, this could be overcome by using drones that obtain accurate geo-located imagery (e.g. using real-time kinematic (RTK)) and very high-resolution sensors with a zoom function. This could allow very accurate counts of *Cassiopea* from drones flying at much higher and safer altitudes (e.g. 50–75 m) than we used in our study. Furthermore, it is possible that the use of multi-spectral or hyperspectral sensors on drones in future research could improve the detectability of jellyfish in more turbid or deeper water [34]. Recent advances in affordable RTK drone with multispectral sensors may provide improved research capacity for monitoring jellyfish. In addition, the legal requirements for drones to remain in visual line of sight will likely relax in coming years with the development of unpiloted aircraft traffic management systems that facilitate safe, beyond visual line of sight drone traffic [55,56].

Survey time and costs are key considerations when deciding between direct and drone-based sampling methods. We showed that the distribution of *Cassiopea* is patchy, hence we recommend five to nine 50 m transects per site (depending on the site’s area) for appropriate precision when making direct visual observations. However, this level of sampling effort is time-consuming and will result in around 135 minutes sampling time, plus the extra time required to unload and load the kayak and associated equipment before and after sampling. A drone, on the other hand, only takes 15 minutes to fly the pre-programmed grid of the whole-site, and, while drones require over twice as much time during the analysis stage in order to review the footage, it is advantageous that the footage can be revisited multiple times, unlike personal observations from a kayak [34]. The linear relationship between the number of *Cassiopea* detected by drones and direct observations demonstrate that direct observations are still a reliable technique and have the added advantage of allowing specimens to be sampled for additional studies such as genetics, morphology, life history, physiology etc. However, when considering purely spatial/temporal density surveys, examining the overall cost and time of the two techniques showed that drones are more efficient as the sampling and analysis cost scale up with time.

The maximum density of *Cassiopea* recorded in this study was 0.15 individuals per m^2^, which is relatively low compared to densities of up to 168 individuals per m^2^ recorded overseas [29]. *Cassiopea* could be experiencing decreased performance in response to the low winter temperatures experienced at their invasion front in temperate Lake Macquarie, limiting their ability to invade in high densities [57]. The densities and locations of *Cassiopea* recorded in this study should be used as a baseline for future monitoring. Additionally, bell-diameter measurements should be recorded using either technique, as studies have suggested that the benthic coverage of *Cassiopea* is an important factor for determining the ecological impact the genus [29].

Overall, we demonstrate that drones are a precise, efficient and practical tool for long-term monitoring of the distribution and abundance of sedentary *Cassiopea* medusae in shallow waters. The use of transect-based abundance estimates (using drones or kayaks) has the risk of overestimating the number of *Cassiopea* occurring at larger scales and tends to return estimates with low precision. Future studies should benefit from our whole-of-site drone methods as a benchmark for recording the abundance and distribution of *Cassiopea* as they expand their range. The efficient and precise nature of drone surveys allows for cost-effective monitoring of spatially dynamic invasions of *Cassiopea* and should be considered for ongoing monitoring programs.

## Supporting information

S1 Table Transect-level data comparing the number of *Cassiopea* detected along a 50m transect by a drone compared to kayak. Secchi depth reading (cm) for each site was also recorded.
(CSV)

S2 Table Site-level results showing the number of *Cassiopea* detected in the whole-of-site drone footage and the area of each site ≤ 60cm. (CSV)

S1 File Example of drone footage used to count *Cassiopea* along a 50m transect at Karignan Creek. (MP4)
